# Giant Aortic Pseudoaneurysm with Stanford Type A Aortic Dissection after Aortic Valve Replacement

**DOI:** 10.1155/2012/473732

**Published:** 2012-02-20

**Authors:** Mertay Boran, Ali İhsan Parlar, Ertay Boran

**Affiliations:** ^1^Department of Thoracic Surgery, Cankiri State Hospital, Cankiri 18100, Turkey; ^2^Department of Cardiovascular Surgery, Trabzon Ahi Evren Training and Research Hospital, Trabzon 61040, Turkey; ^3^1st Department of Anesthesiology and Reanimation, Izmir Ataturk Training and Research Hospital, Izmir 35150, Turkey

## Abstract

Giant pseudoaneurysm of the ascending aorta is a rare but dreadful complication occurring several months or years after aortic surgery. Thoracic aortic aneurysms tend to be asymptomatic and were previously often diagnosed only after a complication such as dissection or rupture. We present a rare case of giant ascending aneurysm with Stanford type A aortic dissection occurring 6 years after aortic valve replacement and also illustrate the potential dimensions the ascending aorta may reach by a pseudoaneurysm and dissection after AVR.

## 1. Introduction

Giant pseudoaneurysm of the ascending aorta after aortic valve replacement (AVR) is a rare and dreadful complication, which may occur several months or years after aortic surgery [1–3]. These aneurysms tend to be asymptomatic and are often diagnosed only after a complication such as dissection or rupture [3, 4]. We present a case of giant ascending pseudoaneurysm with Stanford type A aortic dissection occurring 6 years after aortic valve replacement. The case also illustrates the potential dimensions the ascending aorta may reach by a pseudoaneurysm and dissection after AVR.

## 2. Case Presentation

**Figure 1:**
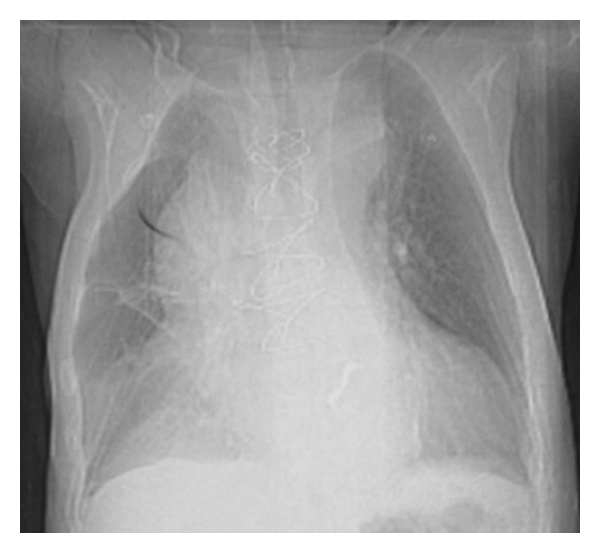
Chest X-ray revealed enlargement of mediastinum.

**Figure 2:**
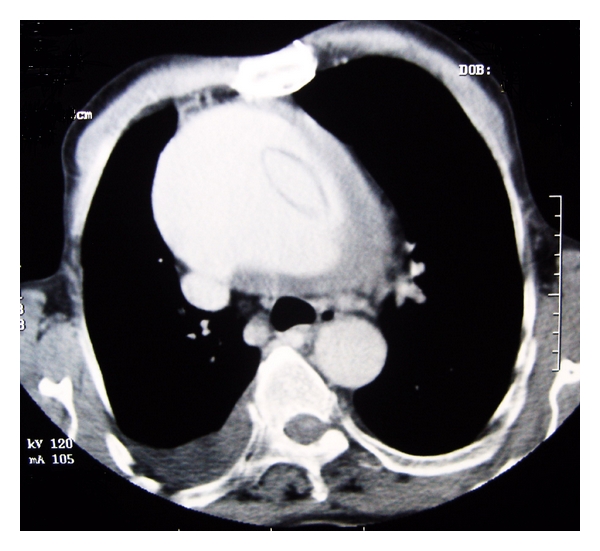
CT scan of the chest with an intimal flap separating the true and false lumen, mural thrombus near the intimal flap, aneurysm of the ascending thoracic aorta approximately 11 cm in diameter, and pleural effusion at right hemithorax.

**Figure 3:**
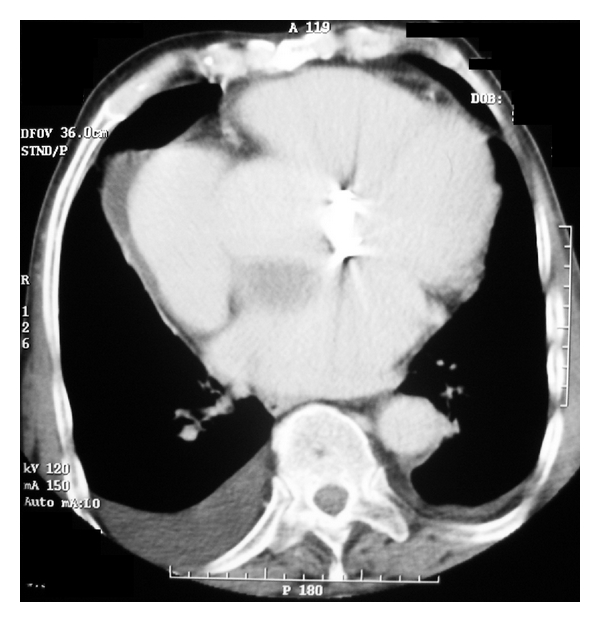
CT scan of the chest revealed pleural effusion at right and left hemithorax and prosthetic aortic valve.

A 71-year-old man, with increasing interscapular and back pain complaint that started 3 days before, was taken to emergency department after a syncope episode. He had a history of aortic valve (bileaflet mechanical heart valve) replacement surgery performed 6 years ago. The patient's physical exam revealed a well-healed median sternotomy incision, 100/60 mmHg of blood pressure, 86 beats/min of heart rate, equal blood pressures in all extremities, and diminished lung sounds at the right lower hemithorax. The laboratory showed only elevated creatinin kinase levels while hemoglobin and hematocrit levels were in normal limits. Chest X-ray revealed sternal closure sutures and enlargement of mediastinum and opacities of prosthetic aortic valve (Figure 1). CT scan of the chest demonstrated an intimal flap separating the true and false lumen, aneurysm of the ascending thoracic aorta approximately 11 cm in diameter, large thrombus surrounding the aorta, bilateral pleural effusion, more at the right (Figure 2) and minimal at the left hemithorax and prosthetic aortic valve (Figure 3). Transthoracic echocardiography showed a markedly enlarged ascending aorta, intimal flap in the aorta, normal prosthetic valve function and 50% ventricular ejection fraction. The patient's previous outpatient control echocardiographies performed 5 and 18 months before revealed normal prosthetic valve function and an enlarged aortic root (4.4; 4.1 cm, resp.) and ascending aorta (5.1; 5 cm resp.). Due to his stable condition, management included only pain therapy and his immediate transportation to cardiovascular surgery center in another hospital. The patient was discharged 3 days after diagnoses of giant ascending aneurysm with Stanford type A aortic dissection, because he had not accepted the emergent operation. Patient died 21 days later at his home.

## 3. Discussion

 Pseudoaneurysm of the thoracic aorta is dilatation with disruption of one or more layers of aorta's wall. Systemic hypertension, the Marfan syndrome, and previous cardiac surgical procedures (cannulation, cross clamp, AVR) are some of the risk factors [5, 6]. Giant pseudoaneurysm and dissection of the ascending aorta after AVR is uncommon. The incidence of acute dissection among patients with significant aortic dilatation following AVR is 27%, whereas the overall incidence of acute aortic dissection after AVR is 0.6% [6, 7]. The interval between valve replacement and dissection may vary from 2 months to 17 years [1, 2, 7, 8]. The growth rate of the ascending aorta dilatation associated with aortic valve disease is thought to be different from that of an aorta dilated spontaneously with a normal aortic valve [3, 7, 9]. The mechanism of pseudoaneurysm after aortic valve surgery is unclear; defect in the aortotomy suture lines, fragility of the aorta, extensive calcification in the aortic wall, iatrogenic trauma due to manipulation during AVR, infection, jet flow that caused the poststenotic ascending aortic dilatation similar to aortic wall disease in aortic stenosis and regurgitation are some of the possibilities [1–7, 10].

Ascending aortic pseudoaneurysms have high morbidity and mortality rates and result in death from dissection or rupture [2, 4, 9, 11]. Untreated acute-type dissection has a 40–60% mortality risk within 48 hours of the event (1% per hour), however, chronic aneurysms tend to become symptomatic or rupture within 5 years [9, 11]. Thoracic aortic aneurysms tend to be asymptomatic, and nonlocalising symptoms with widened mediastinum on the chest X ray may be some of the presentation forms [3, 4]. Therefore, periodical postoperative imaging after AVR, especially in patients with mildly or moderately dilated aortic root, increases the diagnosis of aortic aneurysms, which allowed elective treatment prior to the development of a complication [2–11]. Dissections of ascending aorta are mostly (79%) characterized by retrosternal pain, while dissections of descending aorta are frequent (64%) together with interscapular as well as back pain [3, 12]. 47% of patient with type A dissections have back pain. Patients without anterior symptoms mostly have history of cardiovascular surgery, aortic aneurysm, or diabetes [3, 12–15].

Aortic dissection is frequently associated with hypertension although hypertension is less commonly seen at initial presentation of ascending dissections. 64% of patients with type A aortic dissection do not have hypertension at the time of presentation. Pleural effusion, widened mediastinum, advancing age (age > 70), type A dissection, and syncope are some of variables associated with hypotension [12, 15]. Pleural effusion and enlarged diameter are signs for potential rupture [1–6].

Aortic regurgitation, aortic size, bicuspid aortic valve, fragility of the aortic wall, and systemic hypertension are some of the risk factors for late dissection of the ascending aorta while previous AVR is an independent predisposing factor for these late dissections [2–11]. In patients with thinned and fragile aortic wall even in the absence of markedly dilated aortic root, prophylactic replacement or wrapping of the ascending aorta should be considered at the time of AVR procedure. In addition, elective reoperation has been also recommended for patients with history of previous AVR, and ascending aorta diameter greater than 50 mm [6, 7].

In the present case, the diameter of the ascending aorta has increased to 11 mm 6 years after AVR and giant pseudoaneurysm has been diagnosed after symptoms due to dissection. Patient has had back pain, although the pain due to ascending dissection is expected to be in more anterior location. The aortic size and previous AVR have been supposed as predictors for back pain and late ascending aortic dissection in our case. Patient at present case was not hypertensive at presentation; advancing age, syncope episode, possibilities of aortic rupture, and false hypotension due to aortic arc dissection were possible related factors with nonhypertensive status.

In the following time, the patient has not accepted the emergent operation and has died 21 days after diagnoses of the giant Stanford type A aortic dissection.

In conclusion, an aggressive surgical approach should be performed in patients with a history of AVR and moderate dilatation of the ascending aorta because of the rapid progression of the ascending aortic disease. Early recognition and diagnosis is essential for appropriate medical and consecutive surgical therapy.

## References

[1] Pieters FAA, Widdershoven JW, Gerardy AC, Geskes G, Cheriex EC, Wellens HJ (1993). Risk of aortic dissection after aortic valve replacement. *American Journal of Cardiology*.

[2] Bachet J, Pirotte M, Laborde F, Guilmet D (2007). Reoperation for giant false aneurysm of the thoracic aorta: how to reenter the chest?. *Annals of Thoracic Surgery*.

[3] Erbel R, Alfonso F, Boileau C (2001). Diagnosis and management of aortic dissection: recommendations of the Task Force on Aortic Dissection, European Society of Cardiology. *European Heart Journal*.

[4] Bonnichsen CR, Sundt TM, Anavekar NS (2011). Aneurysms of the ascending aorta and arch: the role of imaging in diagnosis and surgical management. *Expert Review of Cardiovascular Therapy*.

[5] Milas BL, Savino JS (1998). Pseudoaneurysm of the ascending aorta after aortic valve replacement. *Journal of the American Society of Echocardiography*.

[6] Masuda Z, Murakami T, Shishido E, Kuinose M (2008). A rare cause of dissection of ascending aorta after aortic valve replacement. *Asian Cardiovascular and Thoracic Annals*.

[7] Prenger K, Pieters F, Cheriex E, Griepp RB, Marrone (1994). Aortic dissection after aortic valve replacement: incidence and consequences for strategy. *Journal of Cardiac Surgery*.

[8] Modi A, Vohra HA, Kaarne M (2011). Long-term outcome following repair of acute type a aortic dissection after previous cardiac surgery. *Interactive Cardiovascular and Thoracic Surgery*.

[9] Weigang E, Nienaber CA, Rehders TC, Ince H, Vahl CF, Beyersdorf F (2008). Management of patients with aortic dissection. *Deutsches Arzteblatt*.

[10] van de Wetering MLJM, Wagenaar LJ, Bouma BJ, Koolbergen DR (2010). Giant pseudoaneurysm after aortic root replacement. *Netherlands Heart Journal*.

[11] Enseleit F, Grünenfelder J, Braun J, Matthews F, Jenni R, van der Loo B (2010). Formation of pseudoaneurysm after aortic valve replacement without previous endocarditis: a case-control study. *Journal of the American Society of Echocardiography*.

[12] Hagan PG, Nienaber CA, Isselbacher EM (2000). The international registry of acute aortic dissection (IRAD): new insights into an old disease. *Journal of the American Medical Association*.

[13] Tsai TT, Nienaber CA, Eagle KA (2005). Acute aortic syndromes. *Circulation*.

[14] Sharpe BA, Klompas M (2002). Clinical manifestations of acute aortic dissection. *Journal of the American Medical Association*.

[15] Tsai TT, Bossone E, Isselbacher EM (2005). Clinical characteristics of hypotension in patients with acute aortic dissection. *American Journal of Cardiology*.

